# Chylous ascites with lymphatic leakage localization: technical aspects and clinical applications

**DOI:** 10.1186/s12893-022-01619-7

**Published:** 2022-05-06

**Authors:** Chaoxiang Lu, Lei Wang, Qi Gao

**Affiliations:** Department of Neonatal Surgery, Xi’an Children’s Hospital & The Affiliated Children Hospital of Xi’an Jiaotong University, No. 69, Xijuyuan Lane ,Lianhu District, Xi’an, Shaanxi 710003 China

**Keywords:** Chylous ascites, Carbon Nanoparticle suspension, Lymphatic leakage localization, Medium-chain triglycerides, Prolonged fasting

## Abstract

**Background:**

Carbon nanoparticle suspension (CNS) was applied to locate the lymphatic leakage in chylous ascites (CA). However, the flow speed and distance of the CNS were particularly decreased in the following two cases (patient 5 and 6). This study aimed to investigate and improve the flow speed and distance of the CNS via a rat model.

**Methods:**

Seven patients with CA were accepted for surgery in the past two years. Clinical data were recorded. Rats were divided into two groups to confirm the hypothesis regarding whether accepting milk or orally administered food before surgery was the key factor in CA surgery with CNS. The animals were divided into 2 groups: experimental group of 5 rats receiving fat emulsion injection (2 g/kg) 30 min before the operation and control group of 5 rats receiving saline. We analyzed flow speed and distance of the CNS in two groups of rats. The hypothesis established was that CNS movements pattern differ depending on the degree of capillary lymph duct filling. Finally, the late case reconfirmed the hypothesis again.

**Results:**

In animal experiments, the CNS in the preoperative high-fat feeding group moved faster and over a longer distance than that in the control group (0.51 ± 0.09 cm vs. 0.19 ± 0.10 cm, respectively; *p* < 0.05). Based on this, the CNS was applied to the seventh patient, who had been given a diet with a slightly higher fat content 3 days before the operation, and marked improvement with a complete cure was recorded.

**Conclusions:**

The capillary lymph duct was beginning to swell after dietary intake. The dilation of the lymph vessel could make it easier for the CNS to move and reach the leakage.

**Supplementary information:**

The online version contains supplementary material available at 10.1186/s12893-022-01619-7.

## Background

CA is the accumulation of lymphatic fluid with a milk-like appearance in the peritoneal cavity, and its approximate incidence in children is reported to be 1:10 000 births [1]. Approximately 80% of primary CA occurs congenitally in association with lymphatic malformation [2]. Secondary CA may result from lymphatic disruption after abdominal surgery, infections, abdominal malignancy, cirrhosis and malrotation of the intestine [3].

CA is related to abnormalities of the lymphatic system, such as lymphangiomatosis and lymphangiectasia. There are many infants, however, in whom ascitic fluid is found during a routine pregnancy check. Prenatal management of CA consists of dynamic measurement of the amount of ascitic fluid and termination of gestation if it proves necessary. In the postnatal period, the quick and efficient management of CA is difficult. First, conservative measures, including improving patient comfort and correcting the underlying cause, are the cornerstone of therapy, and treatment should be personalized. Reducing the production and flow of lymph and maintaining a good nutritional condition is the goal of conservative measures. Therefore, high medium-chain triglyceride (MCT) [4] and low long-chain fatty acid milk is a kind of ideal treatment. MCT could cure some types of mild CA, but unfortunately, many patients will still worsen over time. Eventually, surgical intervention with peritoneovenous shunting [5], Denver shunt [6], lymphangiography balloon-occluded retrograde abdominal lymphatic embolization [1, 7], or ligation of the lymphatic duct [8] should be considered.

With advancements in nanoparticle characteristics, CNS could be extensively used in operations [9–11]. CNS, which comprises particles with a diameter of 150 nm, can pass much more easily through the lymphatic vessels (endothelial cell gap, 120–500 nm) than through the blood capillaries (endothelial cell gap, 20–50 nm). Given the above theoretical foundation, we first applied CNS to locate the leakage site in CA during surgery in the past two years. However, despite CNS being widely used, there are few reports on how to improve its efficiency. The aim of this research is to find the reason for the short distance and the prolonged time of the flow of CNS in locating the leakage site in CA and to provide a crucial clinical technique.

## Materials and methods

### Data collection and patient characteristics

 This study was approved by the ethics committee of the hospital in which the patients were treated. The children’s guardians were expressly informed about the surgery risks and benefits, and informed consent was obtained. Seven cases were included (4 CNS cases were previously reported [8], Table 1). All the patients were diagnosed with CA based on abdominal cavity fluid analysis and had a systemic examination performed to confirm that there were no other systemic diseases. All infants had peritoneal drains inserted and were fasted on day 1 of admission. Patients 1–4 were given MCT-milk about for one week before the procedures, and on the remaining dates, as per our previous report. All of our surgical procedure types were compatible with those in previous reports [8]. We collected data on demographic characteristics (Table 1), drainage quality and volume, critical care management, infant feeding patterns and outcomes.

**Table 1:** Details for the last 3 enrolled patients are presented

No	Sex	Weight(kg)	Age (M.D)	Congenital	Days 1	Mean drainage Volume	Preoperative eating	Outcome
5	M	6.5	1 m 10 d	Yes	26	110 cc/d	No	Not Cured
6	F	8.6	3 m 11 d	Unclear	31	95 cc/d	No	Partially Cured
7	M	38.5	13 y 9 m	No	44	130 cc/d	Yes	Completely Cured

Note:

Congenital: whether or not CA occurred in the gestation period

Days 1: days before surgery. Age (M.D): age (year, month and day)

Octreotide: all the patients received octreotide treatment before the operation (dose was 1 to 5 µg/kg/h, starting dose 1 ug/kg/h). Preoperative eating: Once a patient decided to undergo the surgical procedures, milk or food intake were freely allowed for three days before the surgery

Outcome

Not cured: the patient’s drainage tubes could not be removed because of the volume of ascites fluid two weeks after the surgery

Partially cured: the drainage tube could be removed, and there was still ascitic fluid, but it did not affect normal feeding (MCT milk or low-fat diet)

Completely cured: patients were fed a normal diet with no ascites (MCT milk or low-fat diet)

### Animal experiments

The in vivo backflow capacity of the CNS was tested by SD rats CA model, rat ligation of the thoracic duct. Our protocol was performed in accordance with the ARRIVE guidelines (Animal Research: Reporting of in Vivo Experiments). Animal experiments were approved by the animal ethics committee of Xi’an Jiaotong University. The animals were obtained from the specific pathogen-free (SPF) Animal Lab of the Faculty of Medicine at the Xi’an Jiaotong University. Ten SD rats weighing 120 ± 10 gr were randomly divided into 2 groups: a control group (group A) and a CNS group (group B). The experimental group received medium and long chain fat (MLC, 2 g/kg) by gastric gavage, and control group was given the same volume of saline (0.9% NaCl) 30 min before surgery. The rats were anesthetized by intraperitoneal injection with 10 wt% chloral hydrate at 0.4 ml/100 g, and then fixed on the experiment table. Retroperitoneal duct lymph exposed through a midline incision. All rats, the lymph duct was highly ligated near the diaphragm on both ends and dissected in the middle. Fenestration was performed for lymph return decompression at near the distal end.

CNS injection (0.2 ml) was performed into the subserosa along the root of the mesentery at one site in all rats. After injection, the syringe was removed, and cotton swabs were placed in the injection site to prevent the CNS from leaking. Immediately after injection, the CNS maximal diameters (L) at the original point were measured. Then, the maximum longitudinal axis length within 5 min for each rat was designated as D. The actual distances of the CNS were D-L (denoted by S). We determined whether the two groups differed in terms of S. When the experiment was finished, all of the rats were sacrificed under anesthesia.

### Statistical analysis

We performed all statistical analyses using SPSS software, version 22.0 (IBM, Chicago, IL, USA). Significant differences were indicated as *p < 0.05. Differences in the mean values were analyzed using the Wilcoxon test.

## Results

**Fig. 1:**
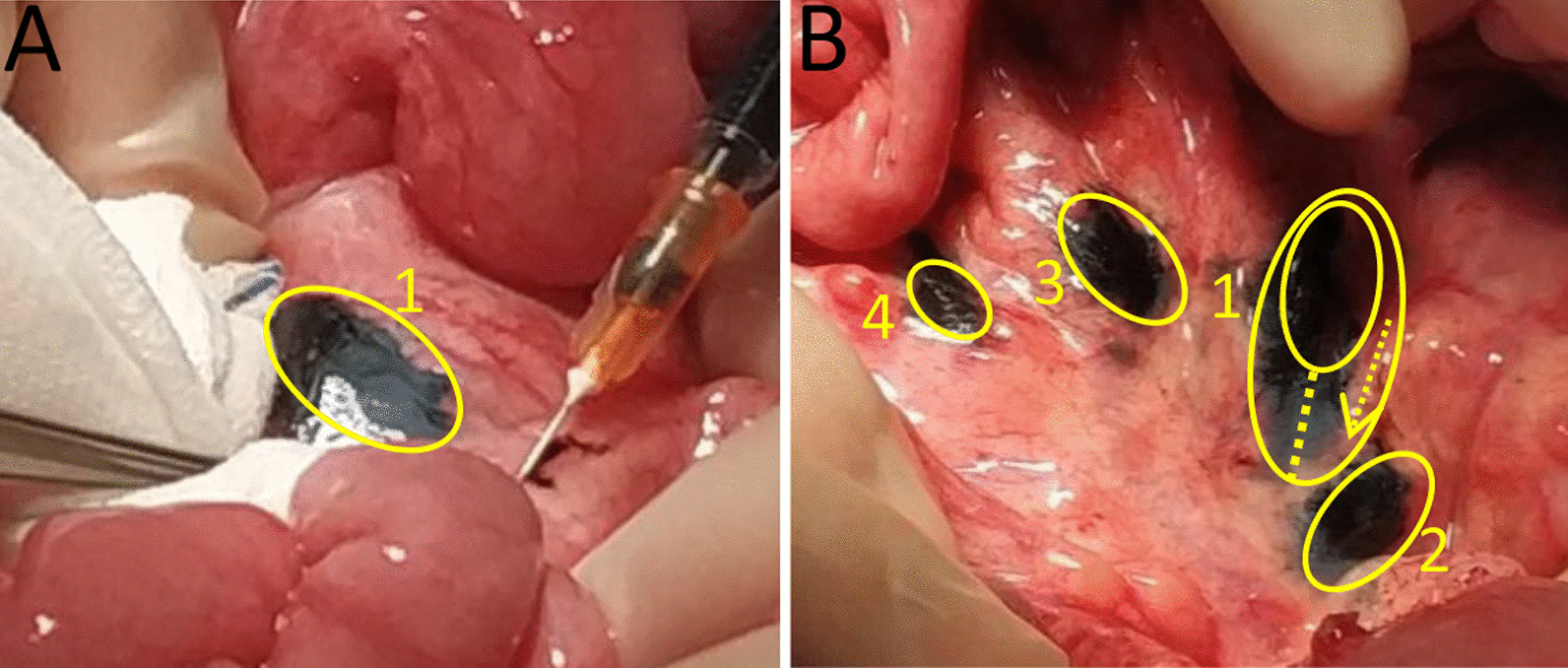
In patients 5 and 6, the CNS moved at a slower rate. **A** Subserosal injection with CNS from distal-to-proximal, sequentially. **B** The first injection shown is labeled as 1. Multiple Injections. Multiple injections of CNS increased the leakage detection rate, with a 5 min interval between each injection. In two cases (case 5 and case 6), injection 1 only moved proximally for a short distance after approximately 20 min

The daily drainage volume over 26 days was close to 100 ml (Table 1) in patient 5 and patient 6. Thus, exploratory laparotomy was performed in these two patients without consistent feeding before the surgery. Multiple injections of CNS increased the leakage detection rate. The maximum diameter were determined after the first injection in the 20th minute again. The discrepancy of two diameters were the distances of CNS. The CNS flowing slower, and the flow distance was significantly shorter in these two patients than those in the previous 4 cases (Fig. 1).

**Fig. 2:**
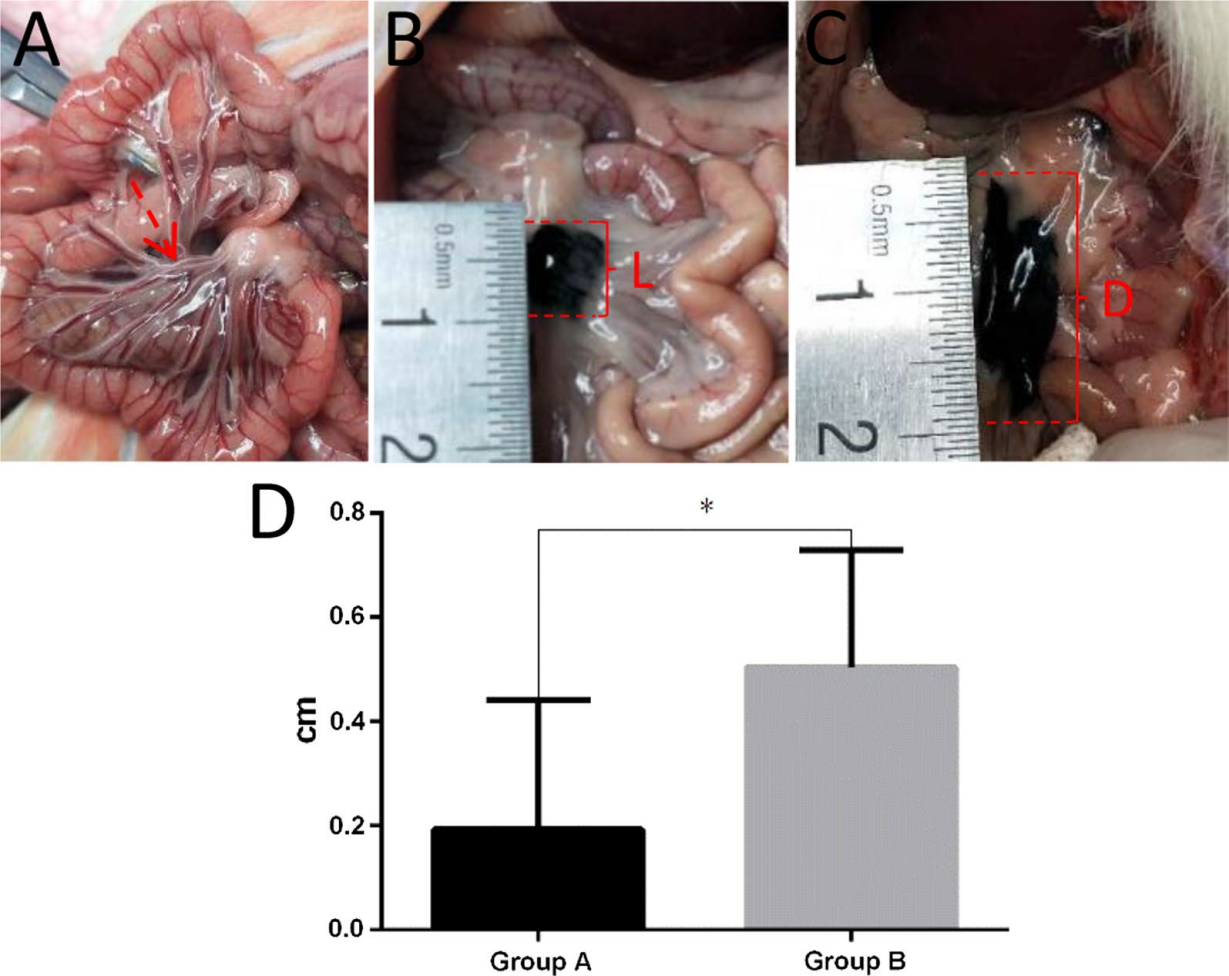
CNS efficacy in the animal model. **A** The mesenteric lymphatic vessels were filled with chylous back flow in group B rats. **B** Rats received subserosal injection of 0.2 ml CNS. The diameter range of the original injection was defined as L (dimensions in cm). **C** Five minutes later, the maximum diameter of the flow range was defined as **D** the difference between D and L was the true distance of CNS movement over 5 min. **D** There was a significant difference between the two groups (*p < 0.05)

All rats completed the full course of the experiment. The average speed of CNS movement in rats in group B was faster, and the moving distance was greater than that in rats in control group A (Fig. 2). Moreover, the mean value of S in the CNS experimental group was 0.51 ± 0.09 cm, which was greater than that in rats in control group A (0.19 ± 0.10 cm, *p* < 0.05).

**Fig. 3:**
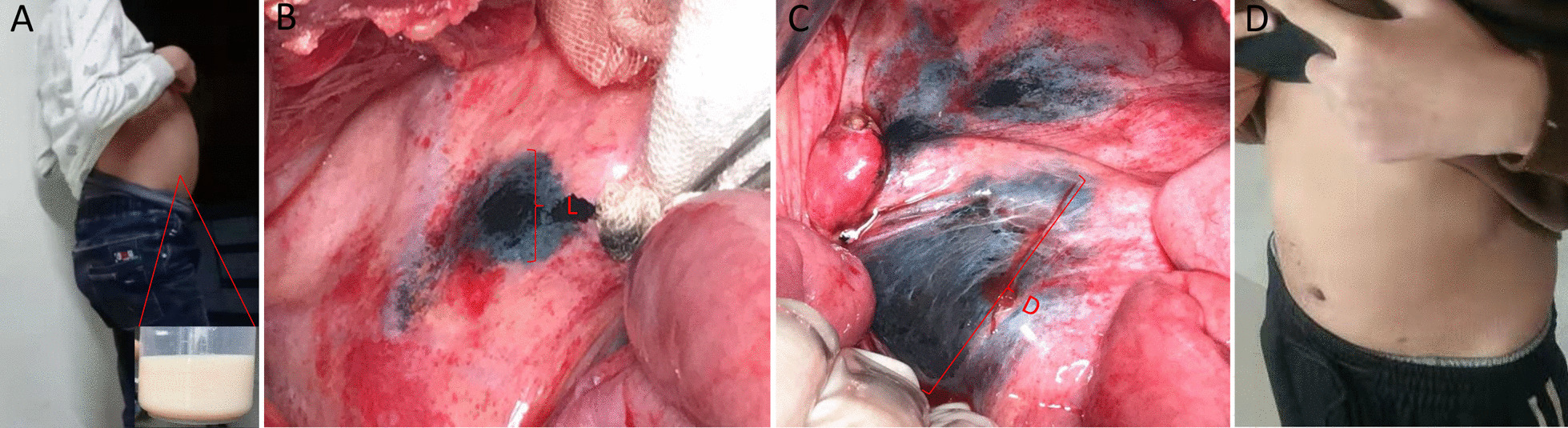
CNS in case 7. **A** Definitive diagnosis is confirmed by abdominal cavity drainage with milk-like ascites. **B** Subsequently, the CNS was injected into the distal lymphatic vessels after ligation. **C** The CNS was refluxed quickly without black fluid leakage. In addition, this finding suggests the lesions achieved full ligation. **D** Two weeks after surgery, abdomen appearance had returned basically to normal

Along with lymphatic vessel filling factors, the seventh patient was administered a normal diet on day 3 before surgery, and the surgical procedure was performed after a 6 h fast. A milky-white liquid continuously flowed out of the root of the mesenteric lymph vessels (Additional file 1: Video S1). The lymphatic leakage was directly ligatured. Remarkably, the leakage was reconfirmed using the CNS, which entered the lymph capillaries from the distal end and exited through the proximal lymphatic leakage site. Compared to those in patients 5 and 6, the velocity of the moving CNS particles was faster and the movement distance was greater in patient 7 (Fig. 3).

## Discussion

In this research, four neonates (previously reported) were successfully treated with CNS localization. The fifth and sixth neonates underwent surgery, during which the CNS moved slowly and only for a short distance. CA was not resolved directly by the surgical intervention. To gain insight into why this occurred, we used a rat experiment to identify and to characterize the correlation between CNS movement and status lymphaticus. By using the data from our recent patient surgery, we have arrived at one major conclusion: for the CA patient with chylous-filled lymphatic vessels, eating before surgery on a routine basis can allow the CNS to move more easily.

Long-chain fatty acids are transformed into monoglycerides and free fatty acids that are then delivered as chylomicrons to the intestinal lymph ducts, contributing to their systemic distribution. By contrast, MCT enter the bloodstream upon absorption and are transported as glycerol and free fatty acids directly into the liver through the superior mesenteric vein. Therefore, the children were given low-fat diets or milk that mainly contained MCT after fasting for 2 weeks. After 2 weeks, the treatment was determined by the amount of ascites fluid. Patients with an amount of ascites fluid notably less than 100 ml could be treated conservatively, whereas for those with at least 100 ml of ascites fluid, an exploratory operation had to be discussed. Surgery should be considered in a patient with an amount of ascites fluid greater than 100 ml over a long period of time or if the amount of ascites fluid increases again to 100 ml in a patient who has been given enteral nutrition. Patients 5 and 6 underwent prolonged fasting without oral feeding before the surgery. Consequently, the role played by the CNS was not completely determined, as described previously by us. A lack of filled lymphatic vessels as a result of fasting before the surgery may explain this observation.

A need for further investigation to confirm this possible theory and to determine the underlying cause was demonstrated by animal experiments. Through this approach, we found that filling of lymphatic vessels 2 h after feeding could make it easier for the CNS to flow through lymphatic vessels to the leakage site. In one of the recent cases, we arranged for the patient to eat freely 3 days before surgery, although a milky white fluid leaked into the peritoneal cavity. Ultimately, consistent with previous cases, the CNS movement was faster and had a longer distance in this case. Thus, the CNS were successfully demonstrated to have the ability to localize to the lymphatic leakage site. Therefore, the experimental and surgery results were consistent with our hypothesis.

The mean size of the CNS is 150 nm, allowing it to easily travel through the lymphatic capillary with an average epithelial gap of 120 to 500 nm but not the blood capillary with an average epithelial gap of 20 to 50 nm. Because of this, CNS has been widely used in diverse fields of surgery [12, 13]. During the surgical resection of a tumor, CNS particles migrate quickly in lymphatic vessels and accumulate in lymph nodes, staining them black. CNS has high specificity for the lymphatic system as a lymph [14] and lymphatic vessel tracer [8]. The CNS moves along the lymphatic vessels to the leakage site within a few minutes after submucosal injection.

The factors that determine the CNS speed are limited, since adults need to be preoperatively fasted for only approximately 6–8 h but not in the long term. Nevertheless, it was remarkable that the degree of filling of the capillary lymphatic vessels remained in good condition. Conversely, a prolonged fasting period is required for the CA patients in the early stage, leading lymphatic vessels not inflated or to epithelial atrophy [15, 16]. As a result, travelling speed of the CNS is very slow in these patients. Thus, we again find that the CNS is effective in locating the leakage position, especially in patients whose lymphatic vessels are filled by food or long-chain fatty acid milk at the right time point.

By utilizing a new strategy to address a complex clinical problem, clinical success was ultimately achieved in 6/7 (85.7%) of patients without any complications or recurrence. The patient can eat during the early stages after surgery instead of undergoing prolonged fasting or a long period of low-fat and medium-chain fatty acid diet consumption. In our most recent case, a less strict low-fat diet was administered 3 times daily beginning on postoperative day 8. In addition, the patient had an uneventful recovery, and no significant ascites fluid was detected. The CNS used in CA shows certain advantages over traditional methods and other types of surgical methods, such as glue embolization, embolization of disrupted lymphatic vessels, splenorenal shunts, indeterminate surgical ligation and peritoneovenous shunting [17–20]. Ultimately, only patients who received definite ligation did not need to pay particularly careful attention to food types. For these patients, vitamin D and other calcium supplements could be regularly taken to prevent calcium deficiency. They were not required to carefully avoid fat-containing foods or agents.

In this report, we have used an objective method to uncover the roles and the impact factor of CNS in CA for determining the leakage position after surgery. Although there were a limited number of cases in our study, our data strongly indicated that filling of the lymphatic vessels is of key importance for CNS functioning. Nevertheless, there were still several deficiencies in this study. First, not all CA patients are suited to undergo ligation. Second, the pathogenesis and the definite cause of the lymphatic leak are still not clearly understood. Therefore, further study is still required.

## Conclusions

The results from this study suggest that the lymphatic vessels are in a low filling state after prolonged fasting, partly explaining the underperformance of the CNS in one particular case. Further research will help to improve the understanding of the mechanism of developmental malformations and to develop more effective interventions.

## Supplementary information

**Additional file 1:** The milky-white liquid flowed out of filled lymph vessels in the seventh patient.

## Data Availability

The datasets generated and/or analyzed during the current study are not publicly available due to patient data confidentiality but are available from corresponding author upon reasonable request.

## Abbreviations

CA: Chylous ascites
CNS: Carbon nanoparticle suspension
MCT: Medium chain fatty acids
